# Supplementary material for: Fast amplitude modulation up to 1.5 GHz of mid-IR free-space beams at room-temperature
Source: Nat Commun. 2021 Feb 5;12:799. doi: 10.1038/s41467-020-20710-2 (PMC7864940; doi:10.1038/s41467-020-20710-2)
Supplement: Supplementary file 1 — Supplementary Information [file 41467_2020_20710_MOESM1_ESM.pdf]

Supplementary Information - Fast amplitude modulation up to 1.5 GHz of mid-IR free-space beams at room-temperature

Stefano Pirotta, Ngoc-Linh Tran, Arnaud Jollivet, Giorgio Biasiol, Paul Crozat, Jean-Michel Manceau, Adel Bousseksou and Raffaele Colombelli

1 Supplementary note 1 - Bare cavity reflectance

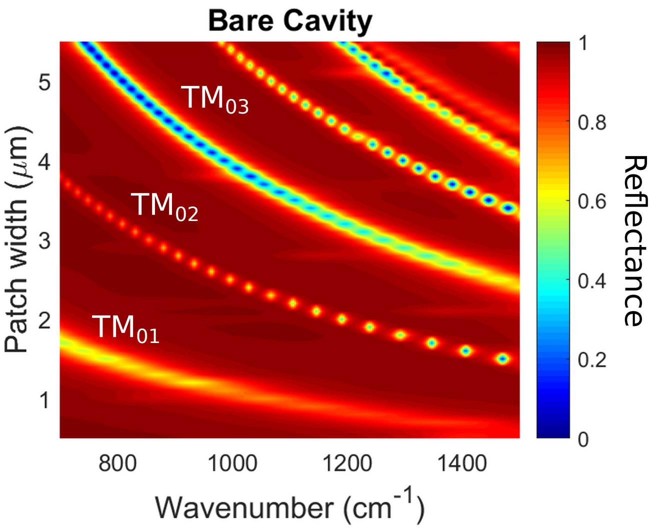

Supplementary Figure 1. Bare cavity reflectance simulations. Reflectance simulated by Rigorous Coupled Wave Analysis (RCWA) for a 1D metallic ribbon bare cavity, as schematized in panels (a) and (b) of Figure 1 of the main text.

Reflectance simulated by Rigorous Coupled Wave Analysis (RCWA) for a 1D metallic ribbon bare cavity, as sketched in panels (a) and (b) of Figure 1 of the main text. The thickness of the insulator layer (GaAs) is 368.1 nm. The effective dielectric function of GaAs with phonon interaction is expressed by:

$$\epsilon_{GaAs} = \epsilon_\infty \left(1 + \frac{\omega_{LO}^2 - \omega_{TO}^2}{\omega_{TO}^2 - \omega^2 - i\omega\gamma_{ph}}\right), \tag{1}$$

where $\epsilon_\infty = 11$ is the relative permittivity at high frequency, γ_{ph} the damping of the phonon mode, $\omega_{LO} = 292$ cm^{-1} and $\omega_{TO} = 268$ cm^{-1} are the longitudinal and transverse optical phonon frequencies, respectively[1].

The metal strip size (patch width, p in Figure 1(b) in the main text) varies from 0.5 μm to 5.5 μm. The distance between the metal ribbons is kept constant at 1.5 μm.

Polarized light, with the electric field orthogonal to the metal ribbons, is directed onto the surface and the is simulated for each width at the incident angle of 10 degree. For this reason the mode TM_{02}, which is non-radiative at normal incidence, is only barely visible. Mode TM_{03} shows the largest contrast[2].

2 Supplementary note 2 - Sample HM4099 reflectance

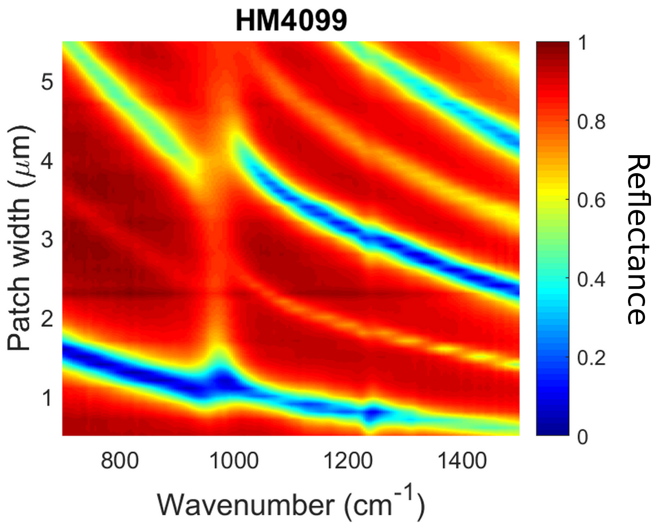

Supplementary Figure 2. Experimental reflectance measurement of the doped sample HM4099 at 300 K.

Experimental reflectance measurement of the sample HM4099 at 300 K. The sample was nominally doped to a sheet concentration of $n_{Si} = 1.74 \times 10^{12} cm^{-2}$. The bare intersubband transition is measured at 955.8 cm^{-1}.

The top metallic grating was implemented by electron beam lithography. The ribbons width varies from 0.5 μm to 5.5 μm and the distance between the metal stripes is 1.5 μm.

The reflectance measurements were performed with a Nicolet FTIR microscope with polarized light (resolution of 8 cm^{-1}) using a liquid nitrogen cooled MCT detector. The Rabi splitting is clearly observed for the TM_{03} mode: this is a clear evidence of the strong light-matter coupling between the cavity mode and the intersubband transition.

The modulator device have been designed based on these measurements, in order to operate on the TM_{03} mode.

3 Supplementary note 3 - Reflectance at 300K of sample HM4098 ($N_{Si} = 6.2 \times 10^{11} cm^{-2}$)

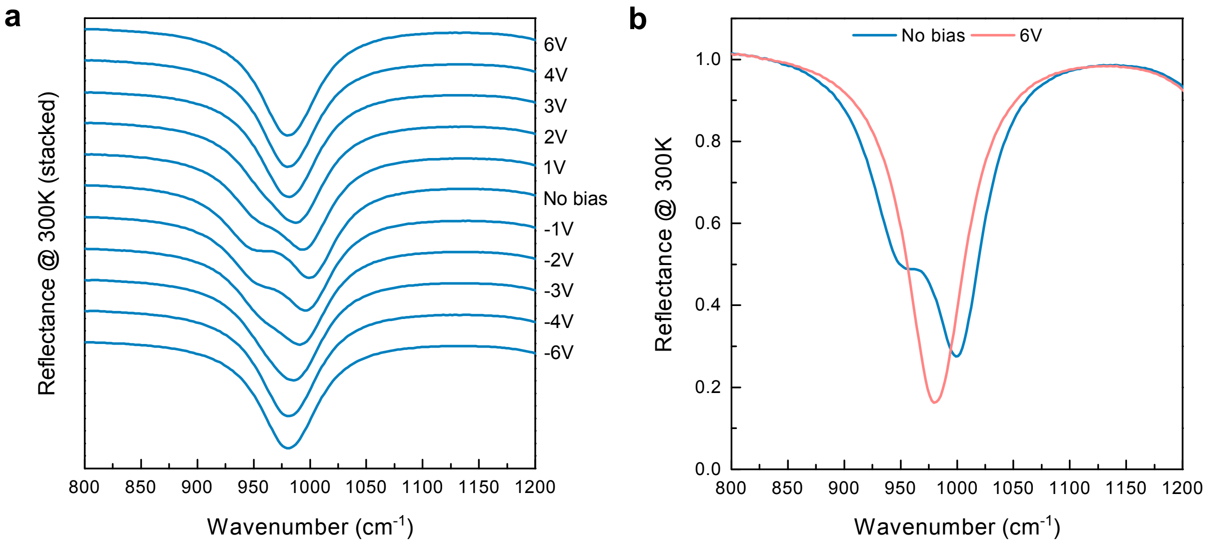

Supplementary Figure 3. 300K reflectance of sample HM4098 under bias (p = 3.8 μm) (a) At different bias (stacked for clarity). (b) Comparison between spectra at no bias (blue solid line) and +6V (orange solid line).

We present here data on sample HM4098 (p = 3.8 μm), that is a lower doped sample. The nominal sheet doping per QW is ($N_{Si} = 6.2 \times 10^{11} cm^{-2}$). In this case the application of a bias completely transitions the system from the strong coupling into the weak coupling regime as confirmed in Figure 3(b). However, the reduced Rabi splitting with respect to sample HM4099, combined with the wavelength range covered by our tunable QC laser, yields a lower reflectance contrast when implemented as a modulator. For this reason, we have employed sample HM4099 to implement the definitive device presented in the main text.

Supplementary references

1. Palik, E. D. (ed.) *Handbook of optical constants of solids* (Academic press, 1998).

2. Balanis, C. A. *Antenna theory: analysis and design* (John wiley & sons, 2016).
